# Lipid Composition but Not Curvature Is the Determinant Factor for the Low Molecular Mobility Observed on the Membrane of Virus-Like Vesicles

**DOI:** 10.3390/v10080415

**Published:** 2018-08-08

**Authors:** Iztok Urbančič, Juliane Brun, Dilip Shrestha, Dominic Waithe, Christian Eggeling, Jakub Chojnacki

**Affiliations:** 1 MRC Human Immunology Unit, MRC Weatherall Institute of Molecular Medicine, University of Oxford, Oxford OX3 9DS, UK; juliane.brun@path.ox.ac.uk (J.B.); dilip.shrestha@rdm.ox.ac.uk (D.S.); 2 “Jožef Stefan” Institute, Jamova c. 39, SI-1000 Ljubljana, Slovenia; 3 Wolfson Imaging Centre Oxford, MRC Weatherall Institute of Molecular Medicine, University of Oxford, Oxford OX3 9DS, UK; dominic.waithe@imm.ox.ac.uk; 4 Institute of Applied Optics, Friedrich-Schiller-University Jena, Max-Wien Platz 4, 07743 Jena, Germany; 5 Leibniz Institute of Photonic Technology e.V., Albert-Einstein-Straße 9, 07745 Jena, Germany

**Keywords:** HIV-1, lipid envelope, lipids, super-resolution, FCS, STED-FCS

## Abstract

Human Immunodeficiency Virus type-1 (HIV-1) acquires its lipid membrane from the plasma membrane of the infected cell from which it buds out. Previous studies have shown that the HIV-1 envelope is an environment of very low mobility, with the diffusion of incorporated proteins two orders of magnitude slower than in the plasma membrane. One of the reasons for this difference is thought to be the HIV-1 membrane composition that is characterised by a high degree of rigidity and lipid packing, which has, until now, been difficult to assess experimentally. To further refine the model of the molecular mobility on the HIV-1 surface, we herein investigated the relative importance of membrane composition and curvature in simplified model membrane systems, large unilamellar vesicles (LUVs) of different lipid compositions and sizes (0.1–1 µm), using super-resolution stimulated emission depletion (STED) microscopy-based fluorescence correlation spectroscopy (STED-FCS). Establishing an approach that is also applicable to measurements of molecule dynamics in virus-sized particles, we found, at least for the 0.1–1 µm sized vesicles, that the lipid composition and thus membrane rigidity, but not the curvature, play an important role in the decreased molecular mobility on the vesicles’ surface. This observation suggests that the composition of the envelope rather than the particle geometry contributes to the previously described low mobility of proteins on the HIV-1 surface. Our vesicle-based study thus provides further insight into the dynamic properties of the surface of individual HIV-1 particles, as well as paves the methodological way towards better characterisation of the properties and function of viral lipid envelopes in general.

## 1. Introduction

**Figure 1:**
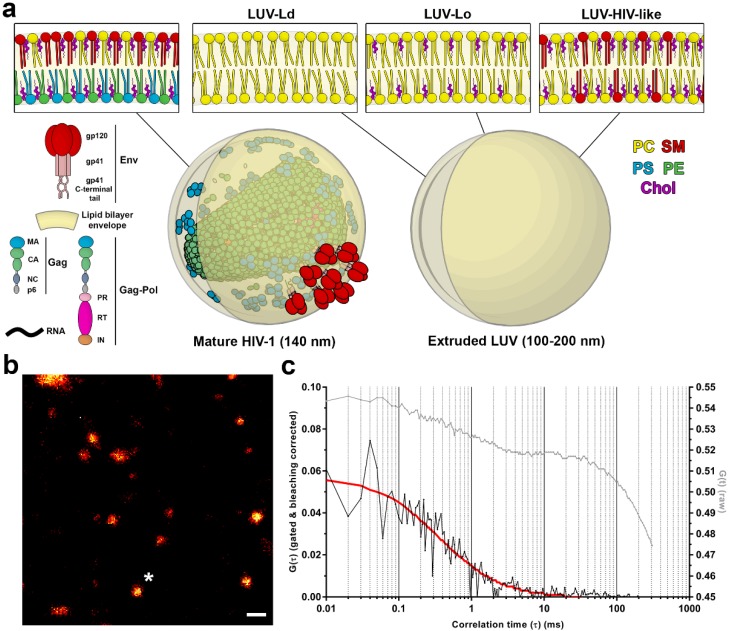
STED-FCS measurements of lipid diffusion in large unilamellar vesicles (LUVs). (**a**) Schematic illustration of lipid and protein composition of mature HIV-1 and 100–200 nm LUVs. HIV-1 Gag and Env proteins and their subunits are indicated along with lipid bilayer components—dioleoyl-sn-glycero-3-phosphocholine ((DO)PC), sphingomyelin (SM), phosphatidylserine (PS), phosphatidylethanolamine (PE), and cholesterol (Chol). HIV-1 lipid composition was adopted from [3]. LUV lipid compositions: DOPC (LUV-Ld), DOPC:Chol at a 67:33 molar ratio (LUV-Lo) or DOPC:Chol:SM at a 37:46:17 molar ratio (LUV-HIV-like). (**b**) Representative STED microscopy image of the extruded LUV-Ld preparation. Imaging was used to locate LUVs of a comparable size and brightness followed by the acquisition of the fluorescence fluctuation data. The white star marks the LUV used for analysis. Lateral fluorescence intensity profiles of representative LUVs are shown in Figure S1. Scale bar: 500 nm. (**c**) Representative raw (grey) and gated & bleaching corrected (black) autocorrelation curves obtained from fluorescence fluctuation data for an extruded LUV. Gated & bleaching corrected autocorrelation curves were fitted using a generic 2D diffusion model (red).

Human Immunodeficiency Virus Type-1 (HIV-1) is an enveloped retrovirus. It acquires its lipid membrane from the plasma membrane of the infected cell during the budding process driven by the assembly of the viral structural protein Gag [1]. In the budded, morphologically mature HIV-1 particle (Figure 1a), this combination of lipids, viral structural proteins, and membrane incorporated viral fusion protein Env and other cellular proteins creates a unique lipid/protein surface environment that is highly curved due to the size (<140 nm) of the virus particle. Lipidomic studies of isolated viral lipids have shown that in comparison to the plasma membrane, the HIV-1 membrane is enriched in sphingomyelins (SMs), glycosphingolipids, cholesterol (Chol), and phosphoinositides, such as phosphatidylinositol 4,5-bisphosphate lipid (PIP2) [2,3]. Such an environment is characterised by a high degree of lipid packing and therefore low polarity within the lipid bilayer. When such a membrane is studied with polarity-sensing probes such as Laurdan, this results in a blue-shifted emission spectrum of the probe [4]. Such spectral changes are often compacted into the General Polarization (GP) parameter, spanning values between −1 and 1: blue-shifted fluorescence results in high GP values, indicating the existence of rigid, highly packed membranes, whereas red-shifted fluorescence results in low GP, signifying fluid, less-packed lipid environments. Laurdan-based spectrophotometric studies of bulk purified particles [5] or spectral scanning microscopy-based analysis of individual particles [6] have indeed shown that HIV-1 membranes have a very high GP value of ~0.5. This indicates a very high level of rigidity that has also been reported in model membranes highly enriched with cholesterol and sphingomyelin [7] or giant plasma membrane vesicles [8]. All these data support the idea that HIV-1 may acquire its lipids and bud from the pre-existing domains of highly packed lipid environment, so called “lipid rafts”. On the other hand, a recent study on supported lipid bilayers suggested that instead of budding from existing lipid rafts, Gag may instead create its own specialised lipid environment, by selectively trapping cholesterol and PIP2 at virus assembly sites [9].

Despite this knowledge of the overall characteristics of the HIV-1 envelope, very little is known about the dynamic characteristics of this environment, such as the mobility of the individual molecules on the surface of individual particles. This is due to the fact that such measurements would require a high spatial and temporal resolution to measure the diffusion of molecules confined within the <140 nm diameter of the virus particles, which is well below the diffraction limit of a conventional optical microscope. Notably, it precludes the application of high temporal resolution spectroscopic techniques such as (confocal) fluorescence correlation spectroscopy (FCS), which relies on evaluating intensity fluctuations arising from fluorophores’ transits through the focused excitation laser beam. However, this shortcoming has recently been addressed thanks to recent advances in the field of super-resolution microscopy. Techniques such as Stimulated Emission Depletion (STED) microscopy can now reach the desired sub-100 nm resolution that is required to image subviral structures [10]. Furthermore, the combination of STED with FCS allows the determination of the molecular diffusion coefficients in the sub-diffraction-sized observation spots [11,12]. In the field of virology, this technique represents a promising opportunity for the study of the molecular dynamics in the context of individual viruses. Recently, STED-FCS in combination with fast line beam-scanning (scanning STED-FCS, sSTED-FCS) has been applied to study the mobility of Env proteins on the surface of HIV-1 particles [6]. This study has established that the HIV-1 envelope is an intrinsically highly immobile environment where Env and other molecules, such as major histocompatibility complex class-I (MHC-I) and glycophosphatidylinositol (GPI)-anchored proteins, all exhibit a very low diffusion coefficient (*D* ≈ 0.002 μm^2^/s). Of note, this mobility is two orders of magnitude slower than that of the same proteins in the cellular plasma membrane [6]. Such a low mobility appears to be due to the combination of the highly ordered and highly curved nature of the viral lipid envelope, the tight packing of internal membrane-interacting virus proteins (MA domain of Gag), and the passive incorporation of many types of cellular proteins during virus budding. Interestingly, the mobility is very low in both immature and mature viruses, despite the differences in Gag organisation (in the case of Env, mobility is even further decreased in immature viruses due to its tight links with the immature Gag shell) [6].

While these findings have provided new insights into previously inaccessible and thus unexplored dynamic aspects of the HIV-1 envelope, there are still many unanswered questions such as whether, similarly to surface proteins, lipids in the HIV-1 envelope also display a reduced mobility, and what the main factors are that may affect their behaviour in highly curved sub-diffraction-sized HIV-1 membranes. Here, we used synthetic lipid vesicles of 100 nm to 1 µm in diameter as a model to examine two such factors: membrane curvature and lipid composition. However, the faster diffusion of lipids compared to membrane proteins required a substantial modification of the experimental approach: instead of the scanning variant [6], we herein applied single-point STED-FCS with a higher temporal resolution. Though this is an established method to measure lipid diffusion properties in large membrane structures, such as giant vesicles or cells [11,13,14], it is (to our knowledge) the first application to small virus-like membrane particles, and the experiments involved the development of a non-standard acquisition and analysis pipeline to combat artefacts resulting, e.g., from significant photobleaching. 

By measuring the mobility of lipids on the surface of vesicles of different sizes and chemical compositions, characterised by different degrees of lipid packing, we find that lipid composition and packing, but not the membrane curvature, play an important role in lowering the mobility of lipid molecules, at least for 0.1–1 µm sized vesicles. This effect may thus contribute to the low protein mobility on the HIV-1 surface. Furthermore, the STED-FCS approach employed to observe the lipid dynamics in small-sized vesicles opens the way for future studies on bona fide HIV-1 and other membrane enveloped virus particles.

## 2. Materials and Methods 

### 2.1. Chemicals

Lipids 1,2-dioleoyl-sn-glycero-3-phosphocholine (DOPC), cholesterol (Chol), egg sphingomyelin (SM), and 1,2-distearoyl-sn-glycero-3-phosphoethanolamine-*N*-[biotinyl(polyethylene glycol)-2000] (DSPE-PEG-biotin) were purchased from Avanti Polar Lipids (Alabaster, AL, USA). The fluorescent lipid analogues Atto647N-DPPE and C-Laurdan were purchased from Atto-Tec GmbH (Siegen, Germany) and 2pprobes (Seoul, Korea), respectively. Bovine serum albumin (BSA) and biotinylated BSA were obtained from Sigma Aldrich (Gillingham, UK), whereas streptavidin was provided by ThermoFisher (Waltham, MA, USA).

### 2.2. Preparation of Large Unilamellar Vesicles (LUVs)

LUVs were prepared from the desired lipid mixture: POPC (LUV-Ld), POPC:Chol (67:33 molar ratio; LUV-Lo), or DOPC:Chol:SM (37:46:17 molar ratio; LUV-HIV-like). For immobilisation on the glass surface and lipid diffusion measurements, lipid derivatives DSPE-PEG-biotin and Atto647N-DPPE were included, each at approx. 1 molecule per 1000 lipids, whereas for GP experiments, C-Laurdan was added at the same concentration. All lipid stock solutions were prepared in chloroform and stored at −20 °C. LUVs of heterogeneous sizes (unextruded LUVs) were formed by drying the lipid mixture by evaporating the organic solvent in a vacuum desiccator for 1 h and rehydrating it in PBS while vortexing vigorously for 10 min, yielding a suspension of approx. 0.5 mM. To obtain LUVs of sizes approx. 200 nm in diameter, part of the heterogeneous vesicle suspension was passed through 200-nm pores (Whatman, Maidstone, UK) 20 times using a manual mini-extruder (Avanti Polar Lipids) preheated to 45 °C. Vesicles were stored at 4 °C and used in experiments within two days.

### 2.3. Preparations of Supported Lipid Bilayers (SLBs)

SLBs were prepared by spin-coating, as previously described [15], using the same lipid mixtures as for LUV preparations, but without the biotinylated lipid and at a lower concentration of the fluorescent lipid probe (1 molecule per 10^4^ lipids). Every solution of lipids in chloroform and methanol (1:1 volume ratio, 1 mg lipids/mL) was spin-coated on a piranha solution-cleaned round 25-mm coverslip (thickness #1.5 by VWR, Lutterworth, UK) for 45 s at 3200 rpm and rehydrated with SLB buffer (10 mM HEPES by Sigma Aldrich, 150 mM NaCl, pH 7.4). Prepared SLBs were kept hydrated in the SLB buffer and used immediately for the measurements.

### 2.4. LUV Immobilisation for STED-FCS Measurements

For microscopy and FCS experiments, LUVs containing the biotinylated lipid were immobilised in eight-well glass-bottomed chambers by ibidi (Martinsried, Germany; glass thickness #1.5), exploiting the biotin-streptavidin-biotin sandwich linker. This approach prevents vesicle flattening and rupture and does not influence their lipid membrane mobility [16]. For this purpose, the chambers were coated with a mixture of BSA and biotinylated BSA (5:1 molar ratio, 1 mg/mL) for 1 h, washed several times with PBS, incubated with streptavidin (500 ng/mL) for 1 h, and then washed with PBS again multiple times. Thereafter, ten-fold diluted PBS suspension of LUVs was added in the prepared chambers for incubation for approximately 30 min. Finally, the non-adhered LUVs were removed by carefully washing the chambers with PBS prior to measurements.

### 2.5. Acquisition of TCSPC Data

Experiments were performed at room temperature using a Leica SP8 STED instrument (Mannheim, Germany) equipped with a 100×/1.4 NA oil immersion STED objective. The lipid probe Atto647N-DPPE was excited by the 633-nm line from the white light laser pulsing at 80 MHz (average power 0.2 or 0.6 μW), depleted with a donut-shaped 775-nm pulsed STED laser (average power 55 mW), and recorded with a hybrid detector in the wavelength range of 640–730 nm. Under these conditions, a 3.2-fold improvement in lateral resolution with respect to confocal images was achieved (estimated from the ratio of transit times of freely diffusing fluorescent lipids in SLBs in confocal vs. STED mode) [15,17,18], resulting in an effective observation spot of approx. 75 nm in diameter (full-width-at-half-maximum, FWHM; see supplementary materials for details). 

Within each sample, vesicles of a comparable brightness and size were selected in confocal images for further measurements of lipid diffusion around 0.1–0.2 and 0.3–1 µm in diameter for the extruded and non-extruded LUVs, respectively (Figure S1 in supplementary materials). Time-correlated single photon-counting (TCSPC) streams from the sites of selected LUVs were acquired for 10–30 s using Hydraharp 400 electronics and SymPhoTime software (both by PicoQuant, Berlin, Germany).

### 2.6. Analysis of STED-FCS Data

The acquired TCSPC data were analysed using FoCuS-point [19] and FoCuS-scan [20] software (Figure S2). First, time-gating was applied to minimise the effects of residual confocal fluorescence and scattered laser light, which would deteriorate the spatial resolution and quality of FCS curves. Next, the resulting heavily decaying time traces were corrected for photobleaching by the local averaging method [20,21]. The calculated correlation functions were then fitted with a 2D diffusion model. From the obtained lateral transit times, diffusion coefficients were calculated using the known diameter of the STED-FCS observation spot (75 nm, see above). Time traces with artefacts due to vesicle movement, and FCS curves with a low signal-to-noise ratio or high fit parameter errors, were discarded based on single-datapoint evaluation, prior to any comparison to avoid bias. 

Please see the supplementary materials for a detailed description of the analysis procedure, together with an in-depth discussion of various technical aspects, such as possible effects of size and curvature in acquisition [22,23] and analysis [6].

### 2.7. Generalised Polarisation (GP) Measurements

Emission spectra of LUV suspensions were measured in transparent-bottom 96-well plates (Porvair, King’s Lynn, UK) by a CLARIOstar microplate reader (BMG LABTECH, Offenburg, Germany), which excited the samples at 385 nm. To calculate the generalised polarization values (GP) of the background-subtracted spectra, fluorescence emission intensities at 440 nm (I_440nm_) and 510 nm (I_510nm_) were used:

GP = (I_440nm_ − I_510nm_)/(I_440nm_ + I_510nm_)

## 3. Results

To demonstrate the strength of the STED-FCS approach in the virological context, we aimed at determining the effect of the membrane curvature and lipid composition on the molecular mobility on the virus-like surface, utilising a synthetic lipid vesicle system. LUVs were generated using either POPC only (LUV-Ld), a POPC:Chol mixture at a 67:33 molar ratio (LUV-Lo), or DOPC:Chol:SM at a 37:46:17 molar ratio (LUV-HIV-like). POPC vesicles represent the highly disordered and fluid lipid membrane, while the POPC:Chol mixture represents a more rigid and ordered lipid composition. The POPC:Chol:SM mixture represents a simplified synthetic HIV-like lipid mixture with a similar GP value and molar ratio of Chol and SM to those found in the real virus [7]. For measurements of lipid mobility in membranes with different curvatures, LUVs of a similar brightness and size were manually selected in images of heterogeneous LUV populations, resulting in two LUV size classes: those with diameters around 0.3–1 µm for non-extruded LUVs and 0.1–0.2 µm for extruded LUVs, respectively (Figure S1 in the supplementary materials). The membrane curvatures of the latter were comparable to those of the HIV-1 viruses (Figure 1a).

To analyse the lipid mobility in each of these conditions, LUVs were doped with a fluorescent lipid analogue DPPE-Atto647N and immobilised on BSA-coated glass coverslips (Figure 1b) using a biotin-streptavidin-biotin sandwich linker, which also prevented the direct contact of the lipids with the glass surface and thereby preserved the diffusion rates of lipids in free-standing membranes [16]. In this study, we used fluorescence correlation spectroscopy (FCS) to investigate lipid diffusion. In FCS, diffusion coefficients of fluorescently-labelled molecules are determined by analysing fluctuations in the fluorescence signal arising as those molecules diffuse in and out of the microscope’s observation spot [24,25]. To realise such measurements on vesicular structures smaller than 250 nm (i.e., smaller than the observation spot of the conventional confocal microscope), we employed FCS on a super-resolution STED microscope generating observation spots <100 nm in diameter (STED-FCS) [11,12]. In our current STED-FCS measurements, the STED microscope was tuned to yield an effective diameter of the observation spot of around 75 nm (see Materials and Methods), thus well below the size of the smallest measured vesicle. Due to the low copy number of Env proteins per individual virus, our previous study of Env mobility on the HIV-1 surface utilised scanning STED-FCS (sSTED-FCS) [6], which minimized photobleaching and enabled the accurate recovery of diffusion coefficients for slowly moving molecules only (such as Env or other proteins in the viral membrane). The number of fluorescent lipid analogues per individual LUV was much higher for our current measurements, thus making photobleaching a less critical issue. Consequently, we used point STED-FCS in this case (with a fixed instead of moving beam during acquisition), offering the high temporal resolution needed to follow the 1000-fold faster diffusion of lipids on the LUV surface compared to protein diffusion in viral membranes. Photobleaching still had to be minimized by using a very low excitation power (10–25-fold lower compared to the sSTED-FCS study [6]). 

Lipid mobility data was acquired for individual fluorescent vesicles of diameters of 100–200 nm (extruded) or 0.5–1 µm (non-extruded) using point STED-FCS in the time-correlated single photon-counting mode (TCSPC). TCSPC mode allowed us to remove non-depleted confocal contribution and residual laser scattering by fluorescence lifetime-based filtering, thus increasing the spatial resolution and signal-to-noise ratio of the acquired signal [26,27]. In addition, photobleaching correction was also applied using a local-averaging method (Figures S2 and S3) [20,21]. The resulting autocorrelation curves (Figure 1c) were fitted with a generic two-dimensional (2D) diffusion model to obtain the average transit times of fluorescent lipids through the sub-diffraction-sized observation spot and to derive the diffusion coefficient for each sample. 

**Figure 2:**
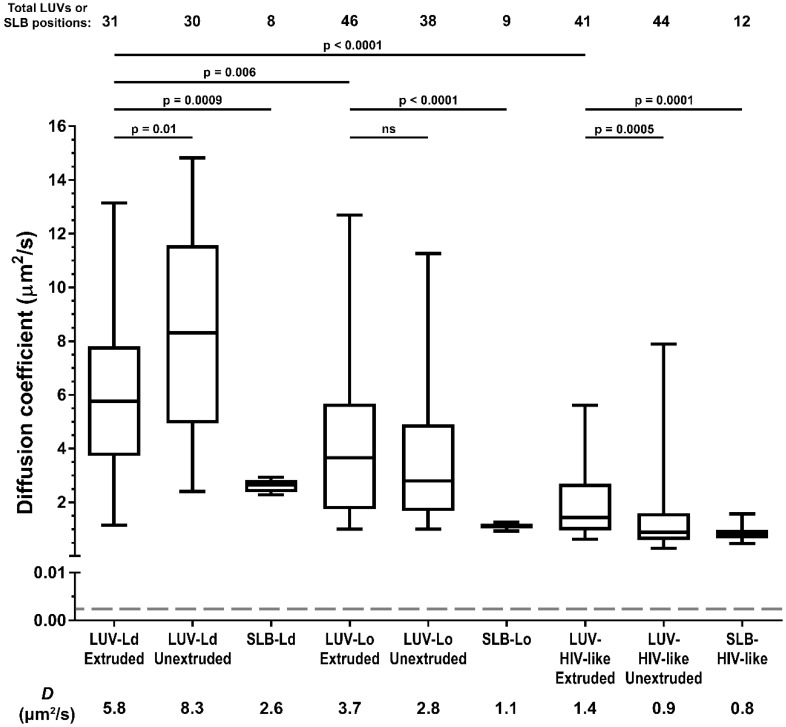
Diffusion coefficients of a fluorescent lipid DPPE-ATTO647N in 100–200 nm (extruded) and 0.3–1 µm (unextruded) large unilamellar vesicles (LUV) and supported lipid bilayers (SLB) with the following compositions: POPC (LUV-Ld, SLB-Ld), POPC:Chol at a 67:33 molar ratio (LUV-Lo, SLB-Lo), and DOPC:Chol:SM at a 37:46:17 molar ratio (LUV-HIV-like, SLB-HIV-like). Median diffusion coefficients (*D*) were determined by STED-FCS measurements of the indicated total number of LUVs and SLB positions from two independent measurements. Results are shown in a boxplot with the following characteristics: horizontal line—median, boxed region—25–75% percentiles or interquartile range (IQR), and vertical lines—min/max measurements. The statistical significance was assessed by the Wilcoxon rank-sum test. The grey dashed line represents the surface Env diffusion coefficient determined for mature HIV-1 particles [6]. Absolute median values of *D* are given below.

Firstly, we compared LUV lipid mobility with the behaviour of the same lipid mixes in supported lipid bilayers (SLB, a lipid bilayer spin-coated onto the microscope cover glass)—a standard model for the measurements of lipid mobility (Figure 2). The results showed that in all LUV formulations, the recorded lipid diffusion coefficients were consistently higher (~2–3 fold) than in the corresponding SLBs with the same lipid composition, as was reported previously [28]. This reduction in lipid mobility observed in supported SLBs compared to free-standing vesicular membranes is caused by Van der Waals interactions of lipid headgroups with the glass surface [29] and not by the incomplete modelling of the molecular diffusion on spherical lipid surfaces of LUVs by a simple 2D diffusion equation (discussed in more detail in the supplementary materials) [6]. SLBs thus do not represent the true zero-curvature control samples in terms of absolute values of diffusion coefficients. Nevertheless, the extracted diffusion coefficients are in good agreement with the values from other independent studies on SLBs and giant unilamellar vesicles (GUVs) [30,31]. Moreover, consistent relative differences between LUV and SLB samples of different lipid compositions indicated that the STED-FCS approach also reliably estimated diffusion coefficients for samples as challenging as LUVs. 

We then compared the impact of the lipid composition and vesicle diameter on the lipid diffusion coefficients in LUVs (Figure 2). Comparison of the diffusion coefficients for 100–200 nm and 0.5–1 µm vesicles for different LUV formulations shows varying relations: the reduction in LUV size caused a decrease in the diffusion coefficient for POPC membranes (*D* = 5.8 µm^2^/s, Interquartile Range (IQR) = 4.1 µm^2^/s, compared to 8.3 µm^2^/s, IQR = 6.6 µm^2^/s), no significant difference for the POPC:Chol LUVs (*D* = 3.7 µm^2^/s, IQR = 3.9 µm^2^/s compared to 2.8 µm^2^/s, IQR = 3.2 µm^2^/s), and an increase for the POPC:Chol:SM mix (1.4 µm^2^/s, IQR = 1.7 µm^2^/s compared to 0.9 µm^2^/s, IQR = 1.0 µm^2^/s). On the other hand, the comparison of the LUVs of the same size but different compositions of increasing rigidity and thus similarity to the HIV-1 lipid envelope (from LUV-Ld to LUV-HIV-like) shows consistent and significant reductions of the diffusion coefficients (*D* = 5.8 µm^2^/s for LUV-Ld, 3.7 µm^2^/s for LUV-Lo, and 1.4 µm^2^/s for LUV-HIV-like in the case of the 100–200-nm sized LUVs). These results suggest the lipid composition of HIV-1 membranes, rather than their curvature, as one of the major factors responsible for the reduction of mobility of the molecules on the HIV-1 surface. 

**Figure 3:**
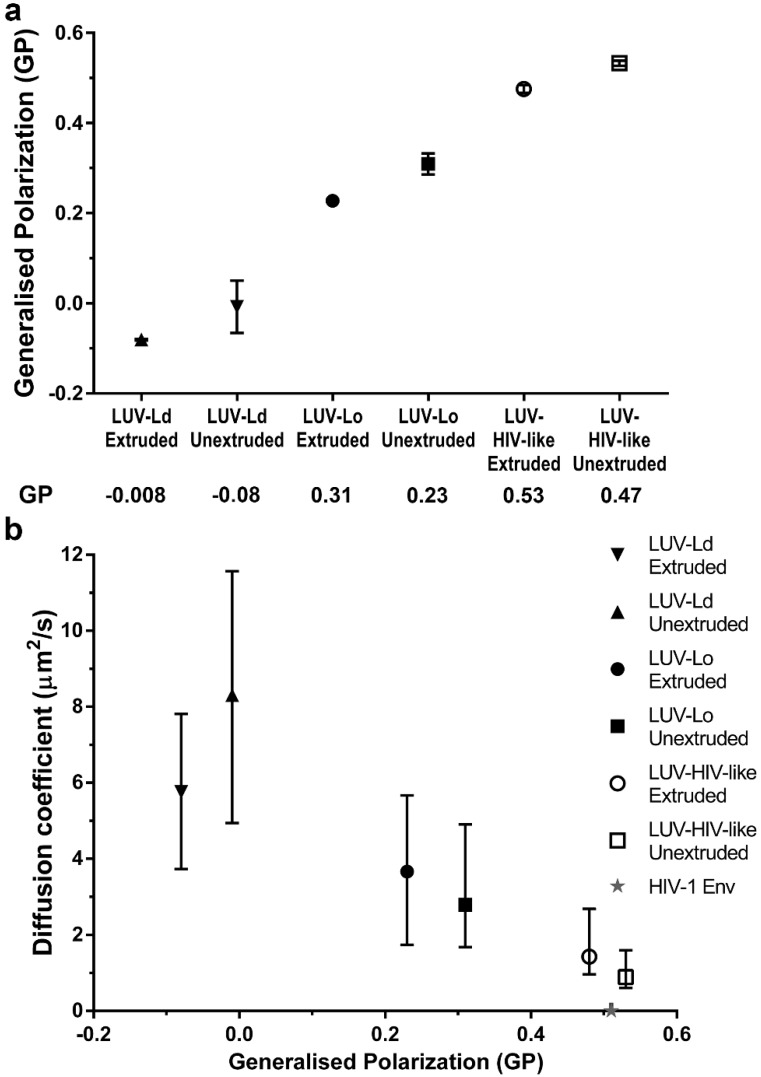
Analysis of LUV membrane properties by C-Laurdan staining. (**a**) Mean ± SD values of the generalised polarisation (GP) parameter were determined for 100–200 nm (extruded) or 0.3–1 µm (unextruded) LUV suspensions with the following compositions: DOPC (LUV-Ld), DOPC:Chol at a 67:33 molar ratio (LUV-Lo), and DOPC:Chol:SM at a 37:46:17 molar ratio (LUV-HIV-like). Results represent data from two independent measurements. (**b**) Median diffusion coefficient vs. average GP values plotted for all LUV sizes and compositions. Error bars represent the interquartile range (IQR) of the LUV diffusion coefficients. The grey star represents the Env diffusion coefficient and GP values in mature HIV-1 particles [6].

To further confirm the importance of increased lipid packing, but not the curvature of the vesicle membranes in the modulation of lipid envelope mobility, we measured the emission spectra of a polarity-sensitive dye C-Laurdan [32] in the membranes of all tested LUV compositions and sizes (Figure 3a). The extracted GP values for POPC vesicles were similar to values reported previously [5]. Moreover, extruded LUVs consistently showed red-shifted spectra and thus lower GP values (~0.07 unit difference) than the corresponding larger unextruded LUVs, indicating, on average, less dense lipid packing in smaller vesicles. However, the differences in GP values were much larger for different lipid compositions than for different sizes within each composition. In fact, increasing GP values (indicative of denser lipid packing) correlated well with the decrease in LUV lipid diffusion coefficients (Figure 3b).

## 4. Discussion

Little is known about the dynamic characteristics of virus surfaces, which is, to a large extent, due to the fact that experimental techniques capable of probing the properties of molecular diffusion with a sufficient spatiotemporal resolution have only recently emerged. Similarly to previous work [6], we herein exploited the combination of the high temporal resolution of FCS with the sub-diffraction spatial scale of the observation spot provided by STED microscopy (STED-FCS). Even if STED-FCS has become a well-established method to study the diffusion properties of lipids and proteins in model and cellular membranes, its application to tiny structures such as virus(-like) particles still presents a considerable experimental challenge, mainly due to low signal levels and rapid photobleaching. For the slow diffusion of proteins, we have previously used scanning STED-FCS [6], which minimised photobleaching at sufficient temporal sampling. Herein, we complemented the toolbox by establishing an efficient workflow to measure the faster diffusion of lipids in virus-like membrane particles by single-point STED-FCS.

This study validated the pipeline on a simpler model system and refined the findings of the previous work that described the low mobility nature of proteins within the HIV-1 lipid envelope [6]. By measuring diffusion coefficients of lipids and the membrane polarity of LUVs of different sizes and compositions, we herein established that the tightly packed nature of the HIV-1 lipid envelope, rather than its high curvature, plays a major role in the creation of a very low mobility environment on the virus particle surface. Considering that the membrane curvature of these particles is, in comparison to the membrane thickness of approx. 4 nm, still relatively mild (see insets in Figure 1a), stronger effects of the curvature are expected, and have indeed been observed, at even smaller sizes of membrane structures—typically below 50 nm [33,34,35], but were nearly absent in less curved membranes [35,36,37]. Though some other methods, such as micropipette aspiration, might be better suited to study diffusion properties in highly curved model membranes, they can induce curvature-driven lipid and protein segregation [38], and, most importantly, cannot be directly applied to native virus-like small membrane structures. To this end, we herein demonstrated that the careful application of STED-FCS can yield meaningful information in a virologically relevant setting.

However, despite observing a four-fold reduction in lipid mobility between most fluid and most rigid LUV compositions, it is clear that this factor is only part of the reason for the even further 700-fold lower molecular mobility of proteins previously observed in bona fide HIV-1 particles (exemplified by a dashed line and a star in Figure 2 and Figure 3b, respectively; unfortunately, we were unable to measure the diffusion of fluorescent lipid analogues in HIV-1 particles, also due to their low efficiency of incorporation). Even accounting for the fact that proteins diffuse approximately 10-fold slower in cell membranes than lipids [6,11], this difference alone is insufficient to explain the extremely low mobility of Env observed in the HIV-1 membrane (*D* ≈ 0.002 μm^2^/s). This discrepancy may be explained by other features of HIV-1 particles that are not present in LUVs, such as tightly packed internal virus structures [39], lipid-MA protein interactions, and the incorporation of Env, as well as a large variety of cellular proteins on the virus surface during the budding process [40]. Such crowded environments knowingly slow down the diffusion rates of both proteins [41,42,43] and lipids [44], and specific membrane-deforming lipid-protein interactions can further enhance this effect [45]. The importance of the above factors on HIV-1 surface lipid mobility can be investigated in future studies employing the STED-FCS approach described here while utilising more sophisticated vesicle models or bona fide HIV-1 particles.

By investigating lipid mobility in small-sized synthetic vesicles, our current study has provided additional insights into the dynamic properties of the HIV-1 surface. It further highlighted the importance of rigid and ordered lipid composition as the contributing factor to the dynamic behaviour of molecules on the HIV-1 virus surface [6], which, in turn, was previously shown to underscore the ability for HIV-1 to successfully fuse with the target cell [10]. Future studies of this still relatively unexplored aspect of the HIV-1 replication cycle may provide a novel therapeutic approach that could potentially prevent virus entry by subtle alterations in lipid packing of the HIV-1 envelope.

## Supplementary Materials

The following are available online at http://www.mdpi.com/1999-4915/10/8/415/s1. Figure S1: Analysis of the size distribution of LUV samples. Figure S2: Exemplary TCSPC histograms, time traces, and FCS curves with fits. Figure S3: Controls for the photobleaching correction of STED-FCS data. Figure S4: Evaluation of out-of-focus contributions.
