# Supplementary material for: Lipid Composition but Not Curvature Is the Determinant Factor for the Low Molecular Mobility Observed on the Membrane of Virus-Like Vesicles
Source: Viruses. 2018 Aug 8;10(8):415. doi: 10.3390/v10080415 (PMC6116177; doi:10.3390/v10080415)
Supplement: Supplementary file 1 [file viruses-10-00415-s001.pdf]

viruses

Supplementary material to the article

Lipid Composition but not Curvature Is the Determinant Factor for the Low Molecular Mobility Observed on the Membrane of Virus-Like Vesicles

Iztok Urbančič[1,2+*], Juliane Brun[1+], Dilip Shrestha[1], Dominic Waithe[3], Christian Eggeling[1,3,4,5*] and Jakub Chojnacki[1*]

[1] MRC Human Immunology Unit, MRC Weatherall Institute of Molecular Medicine, University of Oxford, OX3 9DS, Oxford, United Kingdom
[2] "Jožef Stefan" Institute, Jamova c. 39, SI-1000 Ljubljana, Slovenia
[3] Wolfson Imaging Centre Oxford, MRC Weatherall Institute of Molecular Medicine, University of Oxford, OX3 9DS, Oxford, United Kingdom
[4] Institute of Applied Optics Friedrich-Schiller-University Jena, Max-Wien Platz 4, 07743 Jena, Germany
[5] Leibniz Institute of Photonic Technology e.V., Albert-Einstein-Straße 9, 07745 Jena, Germany
+ Contributed equally to the work.

* To whom the correspondence should be addressed. E-mail: iztok.urbancic@rdm.ox.ac.uk, christian.eggeling@rdm.ox.ac.uk, jakub.chojnacki@rdm.ox.ac.uk

Contents

Resolution of the microscope 2
Quantification of vesicle sizes 2
FCS analysis of TCSPC data 4
Bleaching correction of FCS data 5
Effects of LUV size on acquisition and fitting of the STED-FCS data 6

Resolution of the microscope

The confocal resolution of the microscope was evaluated by analyzing images of immobilized 200-nm Tetraspeck beads (Thermofisher), excited with the 633-nm WLL line, with PSFj plugin for ImageJ [18] (cf. bibliography in the main text), yielding the lateral FWHM of 240 nm (data not shown).

The resolution of STED-FCS was estimated following the standard calibration procedure [15], comparing transit times of freely diffusing fluorescent lipids (Atto647N-DPPE) in SLBs in confocal (τ_{conf}) and STED mode (τ_{STED}):

$$r_{STED} = r_{conf}\sqrt{\frac{\tau_{STED}}{\tau_{conf}}}$$

For POPC and POPC:chol SLBs the transit times τ_{STED}/τ_{conf} obtained by fitting the general 2D diffusion model (see below) were 0.39/3.6 ms and 0.92/9.8 ms (st. dev. ±10%), respectively. These data resulted in the estimated effective observation spot diameters 73 and 79 nm (± 5 nm), respectively, which we rounded to 2 r_{STED} = 75 nm.

Quantification of vesicle sizes

As noted in the main text and clearly demonstrated in Fig. 1b, the obtained vesicles, including the extruded samples, showed a very broad distribution of sizes. To homogenise the populations of the two compared size classes, we individually picked LUVs for FCS measurements based on their similar size and brightness in a confocal image of a larger field of view (Fig. 1b).

To demonstrate the result of this subjective selection, we show exemplary line profiles (Fig. S1a) across six extruded and two non-extruded vesicles of the same composition (LUV-Lo). We estimated the real sizes of the objects (d_{OBJ}) in the approximation that the apparent sizes, determined by fitting a Gaussian function to the profiles, result from the convolution of the (Gaussian) real object with the confocal point-spread-function (d_{PSF} = 2 r_{conf} = 240 nm)

$$d_{OBJ} = \sqrt{d_{IMG}^2 - d_{PSF}^2}$$

obtaining LUV sizes in the range 100–200 nm, which is comparable to the size of HIV viruses (140 nm). For two of these vesicles we also provide the profiles from their STED images (d_{PSF} = 2 r_{STED} = 75 nm), yielding reasonably similar estimates for their sizes as from confocal images. For comparison, we also present intensity profiles across two non-extruded vesicles selected for measurements of the larger class (Fig. S1b), which showed the diameter values around 500 nm.

The presented data therefore corroborate that our subjective selection of extruded vesicles indeed resulted in a population with diameters around that target values, whereas the other class comprised of distinctly larger vesicles (Fig. S1c). By this we excluded the possibility that the small observed differences in the average diffusion coefficients between the two classes were due to a significant overlap of size distributions.

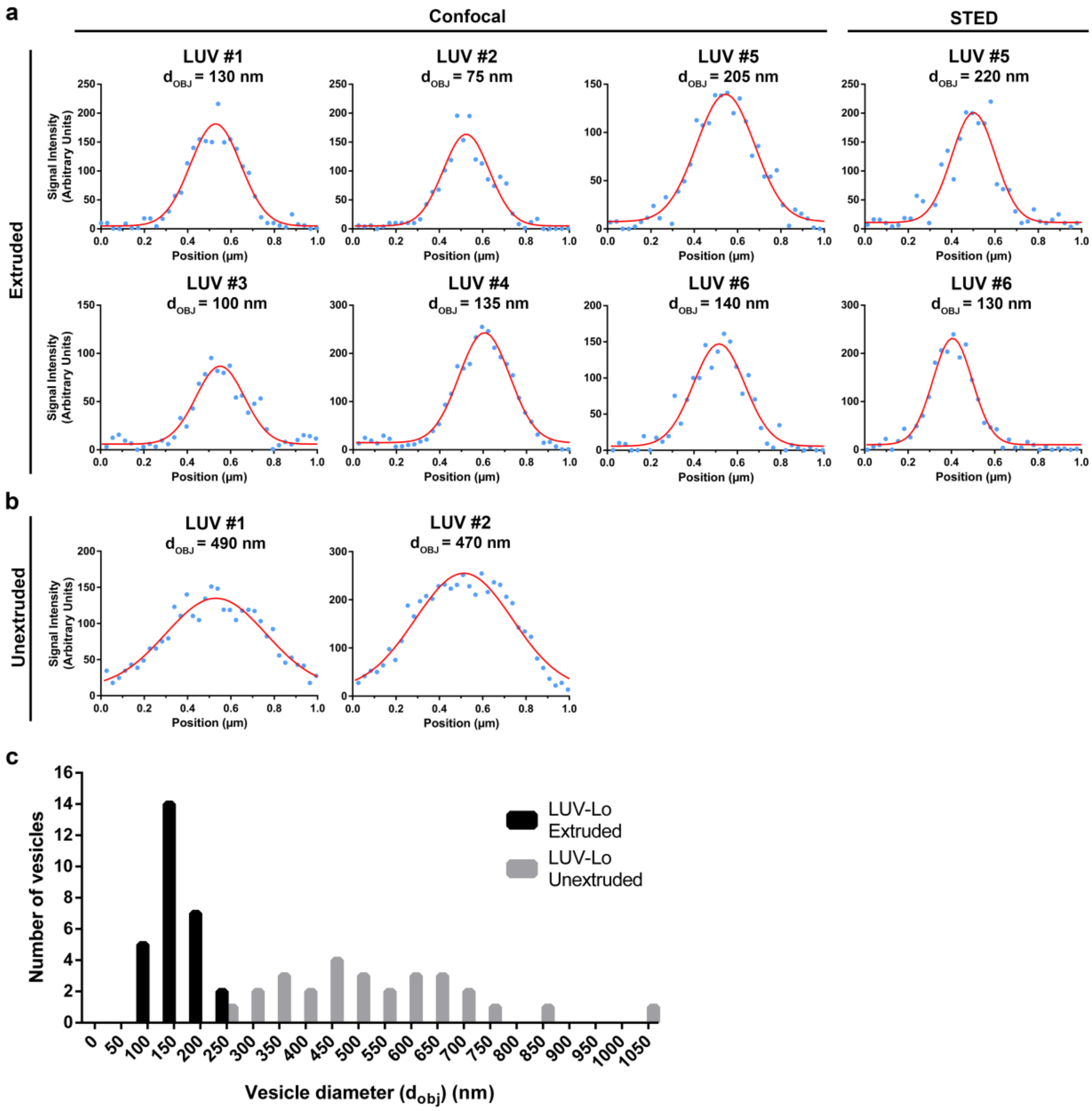

Figure S1. Exemplary intensity profiles of (**a**) six extruded vesicles (LUV-Lo) taken from confocal images, and two from STED microscopy images, as well as (**b**) two non-extruded vesicles of the same lipid composition. The indicated vesicles sizes (d_{OBJ}) were estimated by fitting a Gaussian function (red line) to the intensity profiles (blue dots) and applying the deconvolution approximation, described above. (**c**) Diameter (d_{OBJ}) distribution of LUV-Lo Extruded (black) and Unextruded (gray) vesicle preparations.

FCS analysis of TCSPC data

The recorded TCSPC streams were first time-gated [15] to minimise the effects of confocal fluorescence contribution as well as of scattered excitation and STED light, using FoCuS-point software [19]: from the fluorescence decay histogram only the photons arriving in the 5-ns window immediately after the pulse of the STED laser were selected (Figure S2a) to generate the intensity time traces at a frequency of 100 kHz. These decayed rapidly due to photobleaching of the fluorophores in LUVs (Figure S2b). To minimize the contribution of the background noise, we cropped away the last part of the trace with the signal close to the background level, typically yielding traces of 2–20 s in duration (depending on the excitation power as well as lipid composition and size of LUV), which were then correlated with the FoCuS-scan software [20].

The latter was further used to generate the autocorrelation curves and to fit these with a 2D diffusion model (Figure S2c):

$$G_N(\tau) = C + G_N(0)[1 + (\tau/t_{xy})]^{-1}$$

where $G_N(\tau)$ is the correlation function at time lag τ, C the offset, $G_N(0)$ the amplitude, and t_{xy} the average lateral transit time through the focus of the excitation beam. From the obtained lateral transit times, diffusion coefficients (D) were calculated using the known diameter of the STED-FCS observation spot (2 r_{STED} = 75 nm, see above):

$$D = \frac{r_{STED}^2}{2\ln(2)\,\tau_{xy}}$$

Time traces with artefacts due to vesicle movement, and FCS curves with low signal-to-noise ratio or high fit parameter errors, were discarded based on single-datapoint evaluation, prior to any comparison to avoid bias.

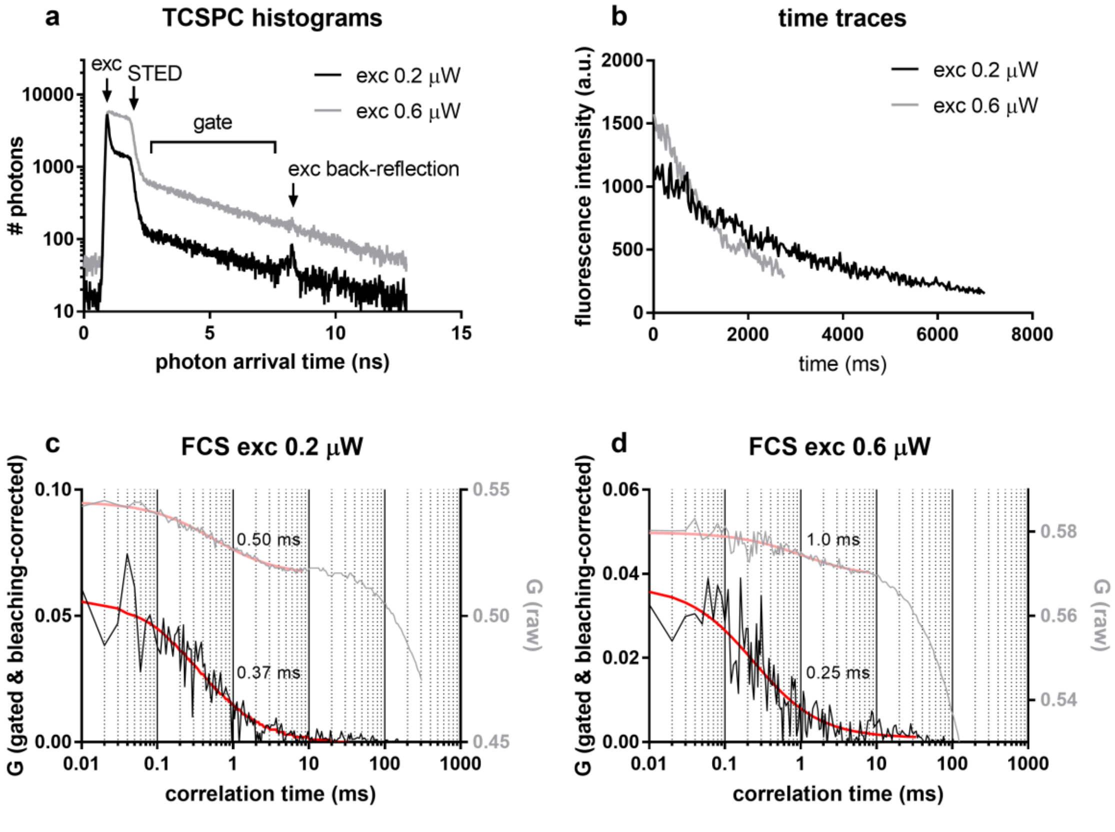

Figure S2. Exemplary data of extruded LUV-Lo acquired at two different excitation powers. **(a)** TSCPC histograms, **(b)** time traces, and **(c,d)** FCS, for which both the raw and bleaching-corrected curves are presented, together with the respective fits to the data and extracted transit times.

Bleaching correction of FCS data

The resulting FCS curves showed a pronounced offset and low-frequency contributions (Figure S2c, top curves), arising from the gradual decay of the average intensity along the course of the acquisition. To correct for these, we used the photobleaching correction with adaptive local averaging (Figure S2c, bottom curves) [21], implemented in the FoCuS-scan software [20]. The method splits the intensity time trace into shorter intervals and averages the resulting autocorrelation functions. In this case we used local-averaging intervals of 0.25 and 0.5 s for faster- and slower-decaying time traces (acquired at excitation powers 0.6 and 0.3 μW), respectively.

We first verified the reliability of the bleaching correction by comparing the results of the same non-photobleaching datasets from the SLBs, analysed with and without the correction, which show no difference (Figure S3a). By applying the correction with different intervals of local averaging to a bleaching dataset of LUV, we further showed that at a short enough interval the fitting errors of individual FCS measurements decreased and the scatter of the dataset of each sample was reduced (Figure S3b). Using these averaging intervals, we obtained offset-free curves with reduced low-frequency contributions (Figure S2c,d with indicated transit times).

This correction method successfully restores the transit times, obtained from measurements with moderate bleaching levels, and thereby enables reliable determination of diffusion coefficients, as we have shown before (also with simulated data) [20]. However, amplitudes of the FCS curves remain affected by the decaying average intensity [21], rendering concentration readouts unreliable and therefore outside the scope of this study. Moreover, this method cannot cope with extreme levels of photobleching, when a large fraction of fluorophores' transits through the excitation beam are subject to photobleaching and hence the transit times are shortened. We avoided this regime by using one of the most photostable dyes on the market (Atto647N) and extremely low excitation powers (10-fold lower than typically used even in confocal FCS). We nevertheless checked for consistency between the data ensemble of the same LUV sample acquired at two different excitation powers that induce vastly different bleaching rates (Figure S3c), confirming the absence of the aforementioned artefacts.

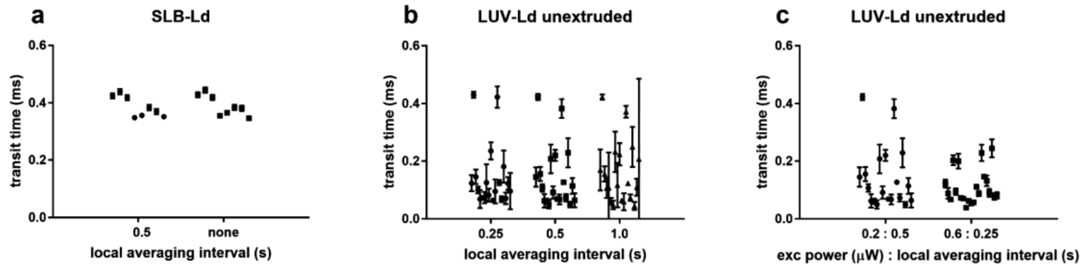

Figure S3. Bleaching controls. Transit times obtained by fitting individual FCS curves, generated: **(a)** with and without the bleaching correction for non-bleaching SLB-Ld samples; **(b)** with local averaging over different intervals for unextruded LUV-Ld; **(c)** from bleaching-corrected data of LUV-Ld acquired at two different powers of the excitation laser. The error-bars represent parameter uncertainties from the fits.

Effects of LUV size on acquisition and fitting of the STED-FCS data

Of note, the applied donut-shaped STED beam narrows down the observation spot only in the lateral direction, leaving the axial component unaffected. Hence, despite focusing on the bottom LUV membrane at the glass surface, some fluorescence signal was inevitably detected also from the top parts of the LUV membrane, especially in the case of extruded LUVs (diameters 100–200 nm). In confocal FCS such an out-of-focus contribution markedly prolongs the observed transit times and dampens the amplitude of the FCS curves [22,23]. In STED-FCS, however, the axial profiles of these two parameters are much flatter around the focal plane (Figure S4) due to the more cigar-shaped effective PSF, which would double the apparent concentration of the probe molecules, but leave their transit time nearly unaffected, preserving the reliability of the evaluated diffusion coefficients. For larger unextruded LUVs (0.5–1 μm), on the other hand, the STED-FCS amplitude of the signal from the out-of-focus membrane is (relatively) even smaller than in the confocal mode, and was, considering the given signal-to-noise level and spread of the data, therefore neglected in the analysis.

To fit our STED-FCS data of LUVs, we used a standard model for diffusion of molecules on a planar surface, which is certainly not a fully accurate description. However, in our previous study of HIV-1 surface mobility we showed that more sophisticated fitting models that consider the diffusion on the surface of a spherical vesicle give comparable results, with a slight tendency towards faster diffusion rates [6]. We would therefore expect similar minor corrections to the results in the present study, which would however not affect our conclusions that the membrane curvature alone cannot explain the very low mobility of proteins observed in the HIV-1.

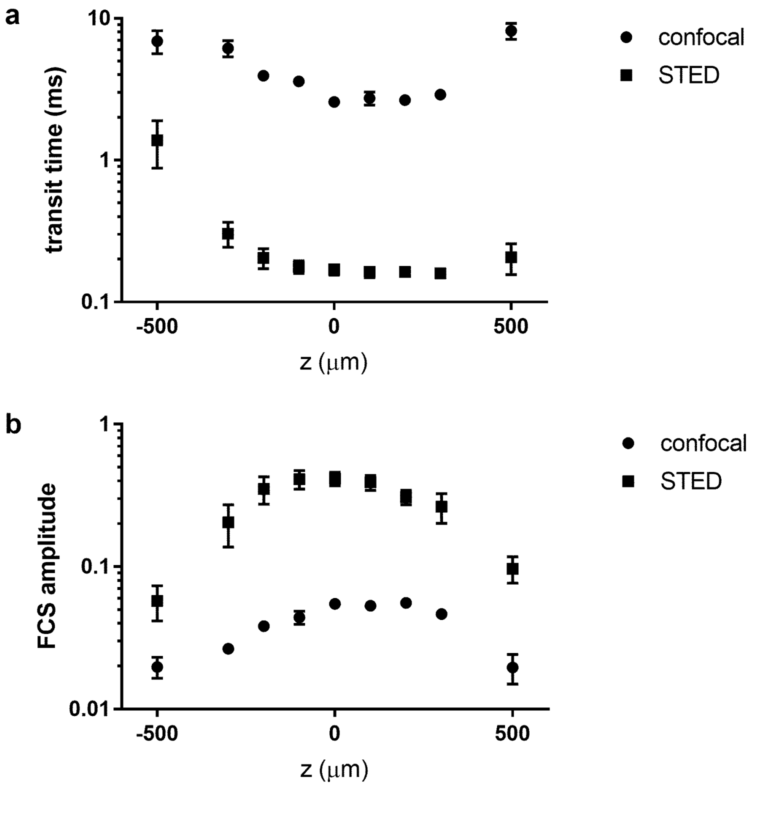

Figure S4. Z-scan FCS recordings of a fluorescent lipid analogue in a supported lipid bilayer (SLB-Ld), performed in the confocal and STED modes. **(a)** Transit time and **(b)** FCS amplitude obtained from fitting the FCS curves acquired at different axial position of the focus with respect to the SLB-Ld in the confocal and STED mode (mean ± st. dev. of at least 3 repeated measurements), demonstrating a lesser dependence of the extracted FCS parameters for the STED measurements.
